# New insights into the genetic diversity of *Schistosoma mansoni* and *S. haematobium*in Yemen

**DOI:** 10.1186/s13071-015-1168-8

**Published:** 2015-10-20

**Authors:** Hany Sady, Hesham M. Al-Mekhlafi, Bonnie L. Webster, Romano Ngui, Wahib M. Atroosh, Ahmed K. Al-Delaimy, Nabil A. Nasr, Kek Heng Chua, Yvonne A. L. Lim, Johari Surin

**Affiliations:** Department of Parasitology, Faculty of Medicine, University of Malaya, 50603 Kuala Lumpur, Malaysia; Department of Medical Laboratories, Faculty of Medical Sciences, Hodeidah University, Hodeidah, Yemen; Azal National Research Center, Azal University for Human Development, 447 Sana’a, Yemen; Department of Parasitology, Faculty of Medicine and Health Sciences, Sana’a University, 1247 Sana’a, Yemen; Parasites and Vectors Division, Department of Life Sciences, Natural History Museum, Cromwell Road, London, SW7 5BD UK; Department of Biomedical Science, Faculty of Medicine, University of Malaya, 50603 Kuala Lumpur, Malaysia

**Keywords:** *Schistosoma mansoni*, *Schistosoma haematobium*, Neglected tropical diseases, Molecular epidemiology, DNA barcoding, Genetic diversity, Evolution, Yemen

## Abstract

**Background:**

Human schistosomiasis is a neglected tropical disease of great importance that remains highly prevalent in Yemen, especially amongst rural communities. In order to investigate the genetic diversity of human *Schistosoma* species, a DNA barcoding study was conducted on *S. mansoni* and *S. haematobium* in Yemen.

**Methods:**

A cross-sectional study was conducted to collect urine and faecal samples from 400 children from five provinces in Yemen. The samples were examined for the presence of *Schistosoma* eggs. A partial fragment of the schistosome *cox*1 mitochondrial gene was analysed from each individual sample to evaluate the genetic diversity of the *S. mansoni* and S. *haematobium* infections. The data was also analysed together with previous published *cox*1 data for *S. mansoni* and S. *haematobium* from Africa and the Indian Ocean Islands.

**Results:**

Overall, 31.8 % of participants were found to be excreting schistosome eggs in either the urine or faeces (8.0 % *S. mansoni* and 22.5 % *S. haematobium*). Nineteen unique haplotypes of *S. mansoni* were detected and split into four lineages. Furthermore, nine unique haplotypes of *S. haematobium* were identified that could be split into two distinct groups.

**Conclusion:**

This study provides novel and interesting insights into the population diversity and structure of *S. mansoni* and *S. haematobium* in Yemen. The data adds to our understanding of the evolutionary history and phylogeography of these devastating parasites whilst the genetic information could support the control and monitoring of urogenital and intestinal schistosomiasis in these endemic areas.

## Background

Schistosomiasis is one of the most prevalent neglected tropical diseases (NTDs) in the tropics and subtropics, where it is endemic in 76 countries. It is estimated that 240 million people are infected, 85 % of which reside in Africa, with nearly 700 million people estimated to be at risk of infection [1–3]. Three *Schistosoma* species, namely *Schistosoma mansoni, S. haematobium* and *S. japonicum* are considered medically important to humans because of their high prevalence rates, pathogenicity and vast distribution [2, 4]. Praziquantel (PZQ) continues to be the drug of choice in terms of controlling the disease in areas with high schistosomiasis morbidity [3, 5]. However, the parasites response to the drug requires suitable monitoring as part of current mass drug administration (MDA) programmes [6].

Pathogen genetic diversity can be affected by many factors, including environmental influence, host immunity and the large-scale administration of treatment [7–14]. High genetic diversity in a parasite population may contribute to the development of drug resistant species and thus cause the emergence of unsusceptible genotypes, although such a correlation is still considered to be somehow controversial [15]. Several studies have investigated the systematics, genetic diversity and structure of *Schsitosoma* species by analysis of the mitochondrial (mt) cytochrome oxidase subunit I (*cox*1) gene [15–20].

Globally, partial *cox*1 analysis of *S. mansoni* isolates has shown a geographical separation into five main groups or lineages, with the most extensive genetic diversity being found in the old world, particularly in East Africa [15, 21]. In contrast partial *cox*1 molecular data of *S. haematobium* showed extremely low levels of genetic diversity within and between *S. haematobium* populations and divided them into two distinct groups; Group 1 was centred around a highly common, persistent and widespread mainland African haplotype (H1) and Group 2 was more diverse and unique to the Indian Ocean Islands [19, 22].

Yemen has been reported to have the highest prevalence of *S. mansoni* and *S. haematobium* in Middle Eastern regions [23]. Despite sustained efforts to control the disease, recent studies have shown a high rate of infection among children in rural areas, whilst also identifying previously unknown transmission foci [24–28]. Despite numerous epidemiological studies on schistosomiais in Yemen, molecular analysis of the *S. haematobium* and *S mansoni* populations has not yet been done. This study was conducted in order to investigate the mt*cox*1 variation of human *Schistosoma* species in Yemen, enabling a better understanding of the genetic diversity and molecular epidemiology of human schistosome in Yemen and the relationship with other geographical populations.

## Methods

### Ethical statement

This study was conducted in Yemen between January and July 2012, after receiving approval from the Medical Ethics Committee of the University of Malaya Medical Centre (Ref. no: 968.4). The study protocol was also approved by the Yemen National Schistosomiasis Control Program (NSCP), the Ministry of Health and Population, as well as Hodeidah University, Yemen.

The parents or guardians and their children were met in their villages where they were invited to participate in this study. A clear explanation of the study’s objectives and methods were given prior to data collection, with written signed or thumb-printed (for those who are illiterate) consent having been obtained from the parents on behalf of their children, and these procedures were approved by the Medical Ethics Committee of the University of Malaya Medical Centre. The children and their parents were informed that they could withdraw from the study at any stage without any consequences and without citing reasons for doing so.

Each participant who confirmed to be infected with schistosomiasis was treated with a single dose of 40 mg/kg body weight of PZQ under observation of the researcher and participating medical officer (Direct Observed Therapy).

### Study design, area and population

**Fig. 1:**
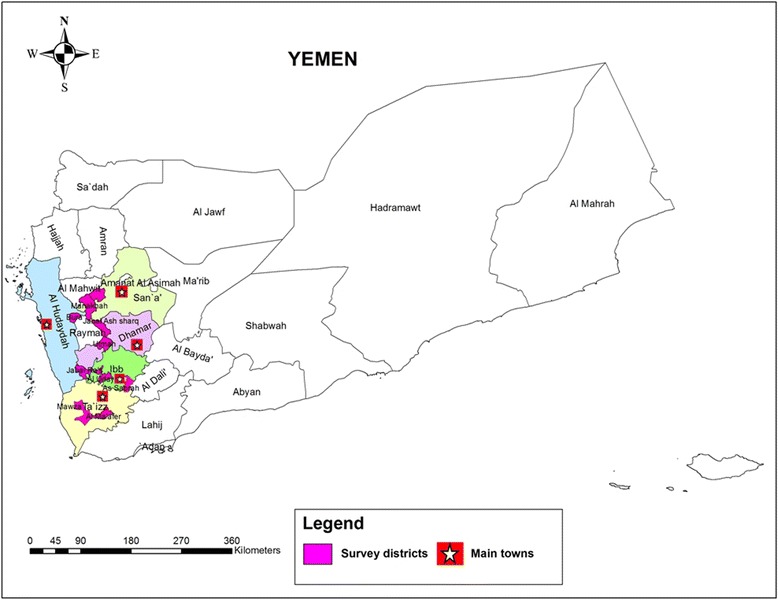
A geographic map showing the location of the districts and provinces involved in the study. The map was created using the Esri ArcMap 10.2.1 software

An exploratory, cross sectional study was carried out among a cohort of children aged ≤ 15 years, all of whom were living in rural communities in Yemen. Data were collected in a period of 7 months from January to July 2012. Overall, 250 households were randomly selected from 20 villages in Taiz, Ibb, Dhamar, Sana’a and Hodiedah provinces. In each province, two rural districts were randomly selected from the available district list and then two villages within the selected districts were considered in collaboration with the Schistosomiasis Control Project office in each province (Fig. 1). The districts were Mosa and Almafer (Taiz), Alsabrah and Alodien (Ibb), Otmah and Gabal al sharq (Dhamar), Alhemah and Manakhah (Sana’a), and Gabal Ras and Bora (Hodiedah). The five provinces are well known as being endemically plagued with both urinary and intestinal schistosomiasis based on information gathered by the Yemen National Schistosomiasis Control Program (NSCP). Of the 632 children who agreed to participate in this study, 400 children successfully submitted the required stool and urine specimens, gave their signed consent and completed the questionnaire (77 from Sana’a, 76 from Taiz, 69 from Ibb, 85 from Hodiedah and 93 from Dhamar).

A description of the study area and population details have been published previously [28].

### Parasitological surveys

Faecal and urine samples were collected separately into individual clearly labelled 100 ml clean containers with wide mouths and screw-cap lids. The samples were collected between 10 am and 2 pm as this is the maximum egg excretion period that was reported by Gray et al. [29]. The containers were placed into zipped plastic bags before being transported (within 6 h of collection) in suitable cool boxes with a temperature of 4–6 **°**C for subsequent examination at the nearest health centre laboratory. The samples were further subjected to microscopic examination to identify the presence and intensity of schistosome eggs. For *S. mansoni* 1 g of each faecal sample was examined using formalin ether sedimentation and Kato-Katz techniques [30, 31]. *S. haematobium* urine samples were examined for haematuria using a dipstick test (Chuncheon, Korea), and then 10 mls of the urine samples were filtered using nucleopore membranes and the filtrate was examined for schistosome eggs [32]. For molecular analysis about 1 g of each stool sample was preserved in 70 % ethanol (DNA-friendly) before being refrigerated [33] and about 1 ml of sediment from each urine sample was preserved in 70 % ethanol before being refrigerated [34]. The preserved specimens were transferred to the Department of Parasitology, Faculty of Medicine, University of Malaya, Kuala Lumpur and kept refrigerated for molecular processing.

### DNA preparation

Prior to DNA extraction each ethanol-fixed stool and urine sample was put in a 2 ml microfuge tube and washed three times in MilliQ H2O buffer. The samples were then centrifuged for 5 min at 2000 rpm to remove the ethanol. Lysis buffer from the DNeasy Blood & Tissue Kit and QIAamp DNA Stool Mini Kit (QIAgen, Hilden, Germany) was then added to each urine and faecal sample. Genomic DNA was extracted from each stool and urine sample according to the manufacturer’s instructions (DNeasy Blood & Tissue Kit and QIAamp DNA Stool Mini Kit (QIAgen, Hilden, Germany).

### Molecular analysis

To detect schistosome DNA in each urine and stool sample, a multiplex schistosome specific PCR was performed using the DNA extracted. The schistosome partial *cox*1 mitochondrial DNA (mtDNA) region was amplified using a universal forward primer ShbmF (5′-TTTTTTGGTCATCCTGAGGTGTAT-3′) with three species-specific reverse primers, ShR (5′-TGATAATCAATGACCCTGCAATAA-3′) for *S. haeamtobium*, SbR (5′-CACAGGATCAGACAAACGAGTACC-3′) for *S. bovis* [35] and SmR (5′-TGCAGATAAAGCCACCCCTGTG-3′) for *S. mansoni* [36]. PCR amplification was performed in 25 μl reactions containing 12.5 μl master mix (QIAGEN Multiplex PCR-HotStarTaq DNA Polymerase), 1.6 μM of the universal forward primer (ShbmF), 0.8 μM of each of the three reverse primers (ShR, SbR and SmR) and 2 μl of DNA (~103.7 ng/μl from urine samples) and 255.7 ng/μl from faecal samples).

PCR cycling conditions were subjected to an initial denaturing step of 95 °C for 3 min, followed by 30 cycles of 94 °C for 30 s, 58 °C for 1 min 30 s and 72 °C for 1 min 30 s, with a final extension of 7 min at 72 °C. Amplicons were visualized and sized on a 2 % agarose gel stained with SYBR^®^ Safe DNA (Invitrogen, Auckland, New Zealand).

### DNA sequencing and *cox*1 data analysis

PCR products were purified using the QIAquick Gel Extraction Kit (catalog. no. 28104; QIAGEN, Hilden, Germany) and sequenced in both directions using a dilution of the universal forward primer and the specific reverse primer that corresponded to the species specific amplicon size (375 bp for *S. mansoni*, 543 bp for *S. haematobium*). Amplicon sequences were run on an Applied Biosystems 3730XL DNA Analyzer (Applied Biosystems, USA) according to the manufacturer’s instructions.

Purified PCR products from samples that showed mixed chromatograms within sequence data were cloned in the pGEM^®^-T Vector (Promega, USA) and amplified in *Escherichia coli* JM109 competent cells. Recombinant clones were selected from each specimen and screened by PCR. Minipreparations of plasmid DNA were done using the QIAprep Spin Miniprep kit (QIAGEN, USA). Three or four clones containing inserts of approximately the expected size were randomly selected for each sample and sequenced according to the method mentioned above.

Sequence Scanner v1.0 (http://www.appliedbiosystems.com) and BioEdit Sequence Alignment Editor Software 7.2.0 (http://www.mbio.ncsu.edu) were used to manually view, edit and remove any sequence ambiguities. Consensus sequences were aligned and any polymorphism between sequences was checked by visualisation of the original sequence chromatograms.

Using the Basic Local Alignment Search Tool (NCBI-BLAST), the consensus sequences were compared and queried to sequence information on the Genbank database to confirm the identity of the species (http://blast.ncbi.nlm.nih.gov). *S. mansoni* and *S. haematobium* sequences were grouped separately and aligned using Clustal W [37]. Any identical sequences were identified and grouped to form individual haplotypes. Individual haplotype sequences were then deposited in the Genbank (Genbank ID: 1783061) (accession numbers: KP294279-KP294306; Tables 1 and 2).

**Table 1:** *S. mansoni* cox1 haplotype polymorphic sites. Each haplotype sequence has been deposited in Genbank

Haplotype code	Genbank accession no.	Variant nucleotide position^a^															
		810	819	831	846	918	921	939	948	972	978	984	988	993	999	1002	1024
Y1ISM	KP294288	C	C	G	T	T	T	A	T	G	G	C	A	G	G	C	G
Y2DSM	KP294289	A	.	A	.	.	.	.	C	.	.	.	G	.	A	.	.
Y3HSM	KP294290	.	.	A	.	.	.	.	.	.	.	.	G	.	A	.	.
Y4TSM	KP294291	.	.	A	.	.	.	.	.	.	A	T	G	A	A	.	A
Y5TSM	KP294292	.	.	A	.	.	.	.	.	.	A	.	G	A	A	.	.
Y6ISM	KP294293	T	.	A	.	.	.	.	.	.	A	.	G	.	A	.	.
Y7TSM	KP294294	.	.	A	.	.	.	.	.	.	A	.	G	.	A	.	.
Y8TSM	KP294295	.	.	A	.	.	.	.	.	.	.	.	G	.	A	.	A
Y9ISM	KP294296	.	.	A	.	.	.	.	.	.	.	.	G	.	A	.	.
Y10ISM	KP294297	.	.	A	C	C	A	.	.	.	A	.	G	.	A	.	.
Y11ISM	KP294298	.	.	A	C	C	A	.	.	.	.	.	G	.	A	.	.
Y12ISM	KP294299	.	.	A	C	C	A	.	.	.	.	.	G	.	A	T	.
Y13ISM	KP294300	.	.	.	C	C	A	.	.	.	.	.	.	.	.	T	.
Y14ISM	KP294301	.	.	.	C	C	A	.	.	.	.	.	.	.	.	T	A
Y15ISM	KP294302	.	.	.	C	C	A	.	.	.	.	.	.	A	.	T	A
Y16ISM	KP294303	.	.	.	C	C	A	.	.	.	.	.	G	.	.	T	.
Y17ISM	KP294304	.	.	.	C	C	A	.	.	.	A	.	.	.	.	.	.
Y18SSM	KP294305	T	T	.	.	C	G	G	.	A	.	T	G	.	A	T	A
Y19DSM	KP294306	T	T	.	.	.	G	G	.	A	A	.	G	.	A	T	.

^a^The nucleotide location is taken from the numbering of the partial mitochondrial cox1 gene of *S. mansoni* (Genbank accession no. NC002545.1)

(.) which indicate nucleotides identity

**Table 2:** *S. haematobium* cox1 polymorhisms between haplotypes. Each haplotype sequence has been deposited in Genbank

Haplotype code	Genbank accession no.	Variant nucleotide position^a^											
		740	743	827	842	875	1007	1037	1118	1163	1184	1193	1197
Y1DSH	KP294279	A	T	T	C	C	G	T	T	G	G	T	G
Y2TDISH	KP294280	.	.	.	.	.	.	C	.	.	.	.	.
Y3DSH	KP294281	.	.	.	.	.	.	C	.	.	A	.	.
Y4HSH	KP294282	.	.	C	T	T	.	.	.	.	A	C	A
Y5HSH	KP294283	.	.	C	T	T	.	.	.	A	A	C	A
Y6HSH	KP294284	.	.	C	T	T	A	.	C	A	A	C	A
Y7SSH	KP294285	.	C	.	.	.	.	C	.	.	.	.	A
Y8SSH	KP294286	.	C	.	.	.	.	C	.	.	.	.	.
Y9TSH	KP294287	G	.	.	.	.	.	C	.	.	.	.	.

^a^The nucleotide location is taken from the numbering of the partial mitochondrial cox1 gene of *S. haematobium* (Genbank accession no. NC008074.1)

(.) which indicate nucleotides identity

### Population genetic analysis

Haplotype sequences of *S. mansoni* and *S. haematobium* were analysed using MEGA 5 (www.megasoftware.net). Neighbour-joining (NJ), maximum parsimony and minimum evolution algorithms using pair-wise distances calculated by the Kimura-2parameter (K2P) method [38], with a 1000 bootstrap value were used to investigate the relationships between the haplotypes [39]. Furthermore, a Maximum Likelihood (ML) analysis with 500 replicates was used to investigate the topology of the trees, prior to a best model (HKY + G) being selected based on the ML in jModeltest 0.1.1 [40].

### Haplotype analysis

Nucleotide divergence was calculated for the *S. mansoni* and *S. haematobium* haplotypes using the Juke-Cantor correction model in DnaSP V5.10 [41]. Reference sequences from Webster et al. [15, 19, 22] were also included in the analysis. This was done by alignment of the unique haplotypes consensus sequences of the present study with the indicated published reference sequences using BioEdit Sequence Alignment Editor Software, and then refined manually to fit with our sequences size (375 bp for *S. mansoni*, 543 bp for *S. haematobium*). A minimum spanning network was also generated in order to estimate genealogical relationships among haplotypes using TCS (http://darwin.uvigo.es/software/tcs.html) software.

### Tests of selection

Selection and neutrality tests were conducted in DnaSP V5.10 to investigate any selection in our mitochondrial *cox*1 data without deviating from natural selection using the McDonald-Kreitman and Tajima’s tests.

## Results

### Prevalence of human schistosomiasis

Of the 400 participants, 127 (31.8 %) were egg-positive for schistosomiasis. Overall, 90 participants (22.5 %) had urogenital schistosomiasis, 32 (8.0 %) had intestinal schistosomiasis and 5 (1.3 %) were co-infected with both *S. haematobium* and *S. mansoni* (Table 3). The highest prevalence of schistosomiasis was reported in Hodiedah (37.6 %), followed by Taiz (36.8 %), whereas Dhamar had the lowest rate of prevalence (19.4 %). With regards to schistosome species, Hodiedah had the highest prevalence (36.5 %) of *S. haematobium* infection followed by Sana’a (33.8 %) while Ibb had the highest prevalence of *S. mansoni* infection (31.9 %). Data on the prevalence, distribution and risk factors of schistosomiasis among the participants has been published [28].

**Table 3:** Numbers of egg-positive and PCR-positive urine and stool samples from the 5 schistosomiasis endemic areas in Yemen^a^

Location	No. examined^b^	Egg positive				PCR positive			
		*S. mansoni*		*S. haematobium*		*S. mansoni*		*S. haematobium*	
		*N*	%	*N*	%	*N*	%	*N*	%
Sana’a	77	1	2.7	26	33.8	1	3.2	19	24.4
Taiz	76	8	21.6	24	31.6	9	29.0	19	24.4
Hodiedah	85	1	2.7	31	36.5	1	3.2	26	33.3
Ibb	69	22	59.5	1	1.5	15	48.4	1	1.3
Dhamar	93	5	13.5	13	14.0	5	16.1	13	16.7
Total	400	37	9.3	95	23.8	31	7.8	78	19.5

^a^Data on the prevalence and distribution of schistosomiasis among the participants has been published [28]

^b^Urine and stool samples

(.) which indicate nucleotides identity

Of the 127 egg-positive samples, schistosome *cox*1 amplicons and sequences were obtained from 31 stool and 78 urine samples (Table 3). Only *S. haematobium* specific (543 bp) amplicons were obtained from the urine samples and *S. mansoni* specific (375 bp) amplicons were obtained from the stool samples. On the other hand, 3 % of stools and 13 % of urines egg-positive samples were PCR-negative. These were retested several times, but *cox*1 amplification remained unsuccessful. Moreover, *Schistosoma* egg-negative samples were also PCR negative.

### *Schistosoma mansoni* population genetics

As the schistosome DNA was extracted and amplified from whole faecal samples the DNA sequences represented the genetic profile from a pooled *S. mansoni* population infecting each individual host. Mixed sequence chromatograms were observed at the polymorphic sites within the mt*cox*1 region amplified, with the chromatograms giving the highest peak being recorded as the haplotype data. These haplotypes will therefore represent the most common haplotype found within the pooled population but observations of the mixed chromatograms within sequence data show that there are many more haplotypes that could not be clearly identified. Moreover, our selection was confirmed by cloning and sequencing of samples that showed mixed sequence chromatograms (5 *S. mansoni* and 2 *S. haematobium*). Among the five localities in Yemen, 19 unique *S. mansoni cox*1 haplotypes were detected from 31 samples. Haplotype distribution varied by location and the highest diversity was observed in Ibb and Taiz (Table 4).

**Table 4:** *S. mansoni cox*1 diversity

Location (Isolates)	Haplotype ID	Genbank accession no.	Haplotype distribution within location/% (*n*)	Haplotype diversity (Hd) ± SD	Nucleotide diversity (π)
Yemen (all)	Hap_(1–19)	-	100 (31)	0.959 ± 0.018	0.02398
Ibb	Y1ISM	KP294288	6.7 (1)	0.952 ± 0.040	0.01858
	Y6ISM	KP294293	6.7 (1)		
	Y9ISM	KP294296	6.7 (1)		
	Y10ISM	KP294297	6.7 (1)		
	Y11ISM	KP294298	6.7 (1)		
	Y12ISM	KP294299	13.3 (2)		
	Y13ISM	KP294300	20 (3)		
	Y14ISM	KP294301	6.7 (1)		
	Y15ISM	KP294302	6.7 (1)		
	Y16ISM	KP294303	6.7 (1)		
	Y17ISM	KP294304	13.3 (2)		
Taiz	Y4TSM	KP294291	11.1 (1)	0.778 ± 0.110	0.00504
	Y5TSM	KP294292	22.2 (2)		
	Y7TSM	KP294294	44.4 (4)		
	Y8TSM	KP294295	22.2 (2)		
Dhamar	Y2DSM	KP294289	80 (4)	0.400 ± 0.237	0.01513
	Y19DSM	KP294306	20 (1)		
Sana’a	Y18SSM	KP294305	100 (1)	0	0
Hodiedah	Y3HSM	KP294290	100 (1)	0	0

*n* = number of samples with that haplotype

**Fig. 2:**
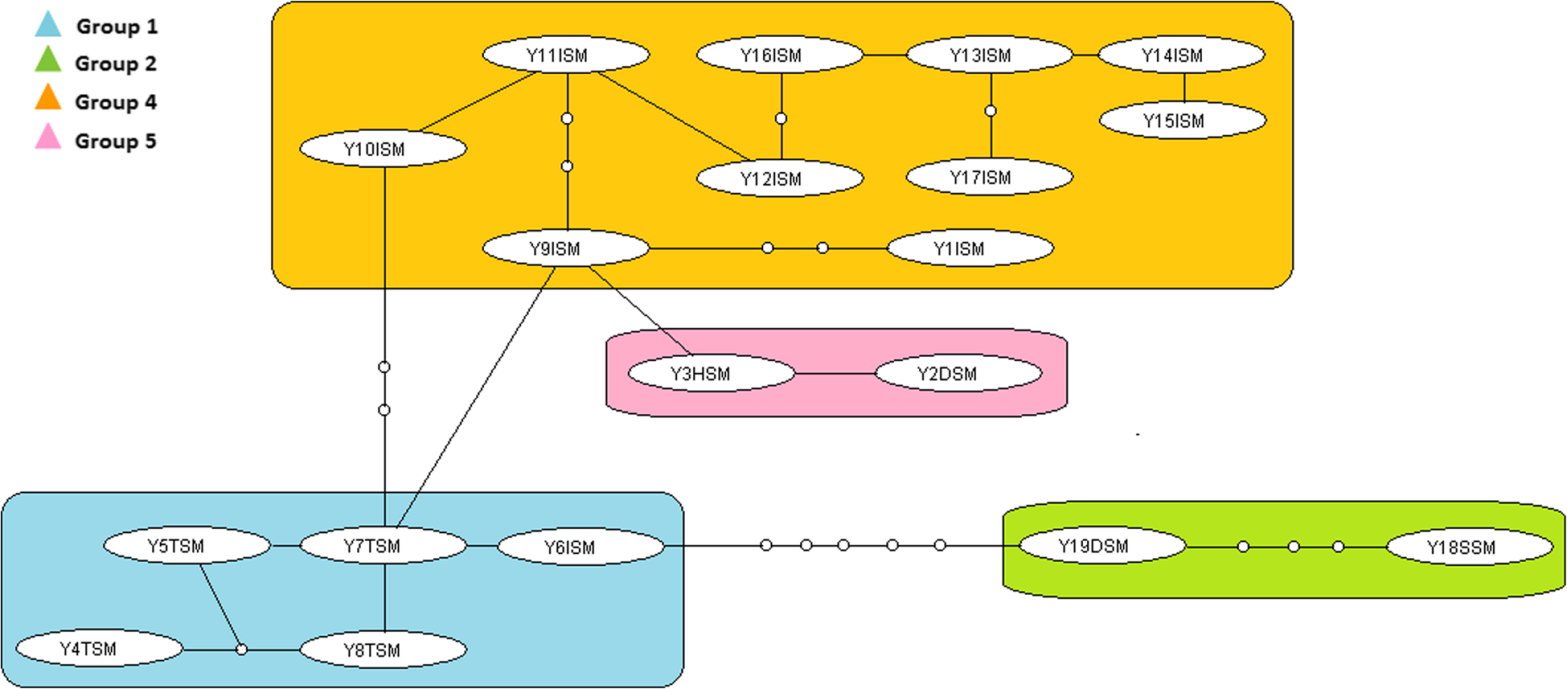
Minimum spanning TCS networks incorporating all 19 *S. mansoni cox*1 haplotypes from Yemen. Each line between haplotypes represents a single bp change and small circles between lines represent unsampled or extinct haplotypes. Group 1: Taiz (YTSM) & Ibb (YISM); Group 2: Sana’a (YSSM) & Dhamar (YDSM); Group 4: Ibb (YISM) Group 5: Ibb (YISM), Hodiedah (YHSM) & Dhamar (YDSM). Grouping of haplotypes was based on Webster et al. [15]

**Fig. 3:**
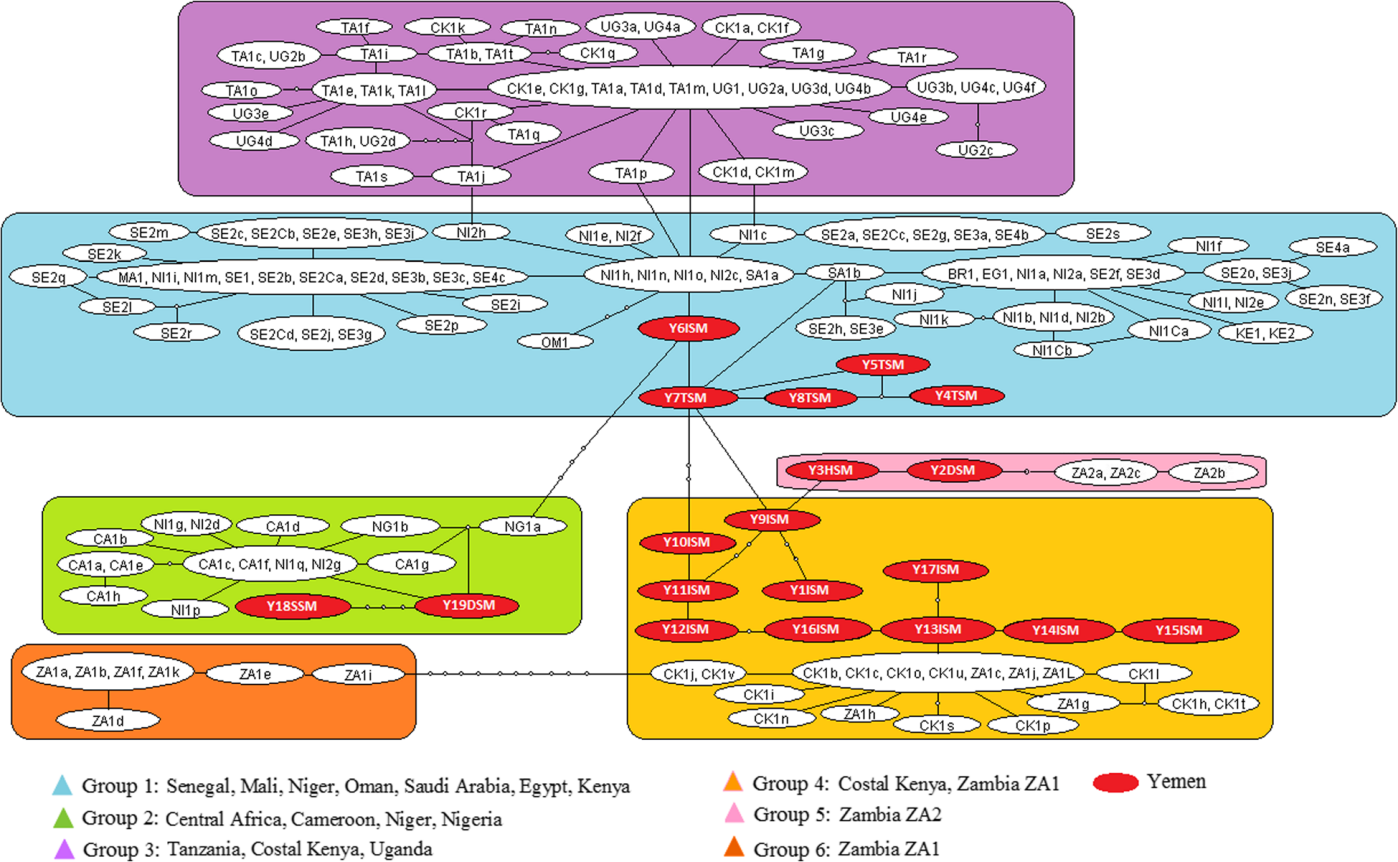
Minimum spanning TCS networks joining the 19 *S. mansoni cox*1 Yemeni haplotypes from this study with other haplotypes from 14 countries within sub-Saharan Africa from Webster et al. [15]. Each line between haplotypes represents a single bp change and small circles between lines represent unsampled or extinct haplotypes. Connecting group 6 with group 4 was done based on a connection limit of 20–30 nucleotide differences. Majority of Yemeni isolates were grouped closely to coastal Kenya and Zambia (group 4) while five haplotypes were linked with more complicated network to Niger, Saudi Arabia, Senegal, Mali, Oman, Egypt and Kenya (group 1). Four haplotypes divided equally between Zambia ZA2 (group 5) and central Africa, Cameron, Niger and Nigeria (group 2). Yemeni haplotypes linked African haplotypes with long branches within four groups

Figure 2 shows the minimum spanning TCS haplotype network for *S. mansoni*. The network consisted of four linked groups but these were not divided by location, therefore there was no population structure observed between different areas. At a geographical level, the minimum spanning TCS network of *S. mansoni* is shown in Fig. 3. The *S. mansoni* haplotypes from the Yemen provinces connected closely to 3 of the 6 geographical groups found by Webster et al. [15]. These haplotypes connected groups 4 (Coastal Kenya and Zambia), 5 (Zambia ZA2), and 2 (Nigeria, Niger and Central Africa) which were connected to Group 1 (Far West Africa, Egypt, Saudi Arabia and Oman). On the other hand, none of the Yemeni haplotypes occurred among Group 3 (East Africa) and Group 6 (Zambia ZA1). Haplotypes found within Ibb province had the highest diversity and were found in 3 of the groups and the Taiz province haplotype (Y7TSM) also provided another link with Group 1.

### *Schistosoma mansoni* phylogenetic structuring

**Fig. 4:**
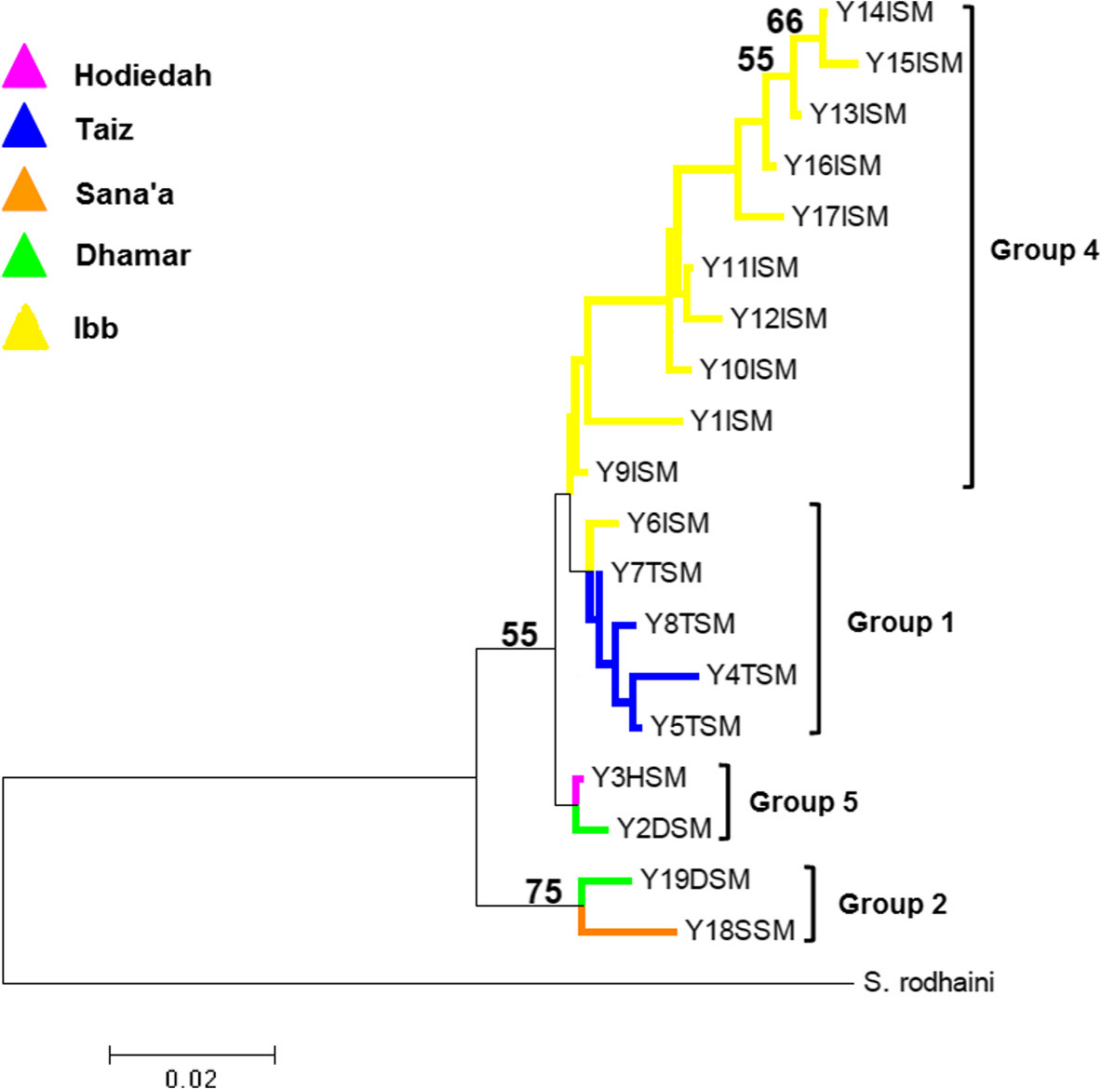
Neighbor-joining *cox*1 phylogenetic tree for *S. mansoni* with 1000 bootstrap values. Nineteen haplotypes clustered into five groups

The *S. mansoni* haplotypes clustered into four groups that correlated to the haplotype network groups, with a clear separation of group 2 from the rest of the haplotypes (Fig. 4). This was also highlighted in the net nucleotide divergence between the groups showing a relatively low genetic separation of the Ibb, Taiz and Hodiedah lineages (groups 1, 4 and 5) compared to the larger divergence found in the Sana’a and Dhamar lineages (Group 2) (Table 5).

**Table 5:** Matrices of net evolutionary divergence (Dxy), between the 5 groups/lineages found in the phylogenetic analysis of *S. mansoni* haplotypes and the out-group sister species *S. rodhaini* ± standard deviation

Groups	G1	G2	G4	G5
G1	-	-	-	-
G2	0.03872 ± 0.01497	-	-	-
G4	0.03101 ± 0.00737	0.04216 ± 0.01250	-	-
G5	0.01514 ± 0.00544	0.04108 ± 0.01665	0.02771 ± 0.00722	-
*S. rodhaini*	0.14218 ± 0.05692	0.14665 ± 0.07335	0.15733 ± 0.05210	0.13923 ± 0.06042

### *Schistosoma haematobium* population genetics

As the schistosome DNA was extracted and amplified from whole urine samples the DNA sequences represented the genetic profile from a pooled *S. haematobium* population infecting each individual host. Mixed sequence chromatograms were observed in only 2 of the sequences indicating that the haplotypes give a good representation of the diversity found within the *S. haematobium* populations. From the five provinces, 9 unique *S. haematobium cox*1 haplotypes were detected within the 78 amplified samples. Diversity was low within and between localities and there was one dominant haplotype (Y2TDISH), which was detected in three out of the 5 provinces, namely Taiz, Dhamar and Ibb (Y2TSH, Y2DSH and Y2ISH), representing 29.5 % of the total haplotypes observed (Table 6). The rest of the haplotypes were unique for their location, with higher diversity being observed in Dhamar and Hodiedah.

**Table 6:** *S. haematobium cox*1 diversity

Location (Isolates)	Haplotype ID	Genbank accession no.	Haplotype distribution within location % (n)	Haplotype diversity (Hd) ± SD	Nucleotide diversity (π)
Yemen (all)	h (1–9)	-	100 (78)	0.819 ± 0.022	0.00911
Dhamar	Y1DSH	KP294279	30.8 (4)	0.641 ± 0.097	0.00143
	Y2DSH (Y2TDISH)	KP294280	53.8 (7)		
	Y3DSH	KP294281	15.4 (2)		
Hodiedah	Y4HSH	KP294282	15.4 (4)	0.615 ± 0.063	0.00252
	Y5HSH	KP294283	30.8 (8)		
	Y6HSH	KP294284	53.8 (14)		
Taiz	Y2TSH (Y2TDISH)	KP294280	78.9 (15)	0.351 ± 0.111	0.00068
	Y9TSH	KP294287	21.1 (4)		
Sana’a	Y7SSH	KP294285	5.3 (1)	0.105 ± 0.092	0.00020
	Y8SSH	KP294286	94.7 (18)		
Ibb	Y2ISH (Y2TDISH)	KP294280	100 (1)	0	0

*n* = number of samples that had the same haplotype. Y2TDISH is the common haplotype found between regions

**Fig. 5:**
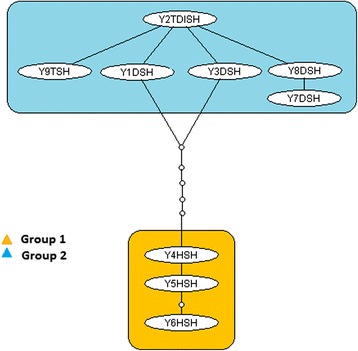
Minimum spanning TCS networks incorporating all 9 *S. haematobium* cox1 haplotypes from Yemen. Each line between haplotypes represents a single bp change and small circles between lines represent unsampled or extinct haplotypes. The network forms 2 groups of haplotypes linked together. Group 1 forms one branch containing only samples from Hodiedah (YHSH). Group 2 forms simple network containing Taiz (YTSH), Dhamar (YDSH), Sana’a (YSSH) and Ibb (YISH). The majority of the samples are closely clustered around the main haplotype (Y2TDISH) with separate single links representing a single polymorphic mutation

**Fig. 6:**
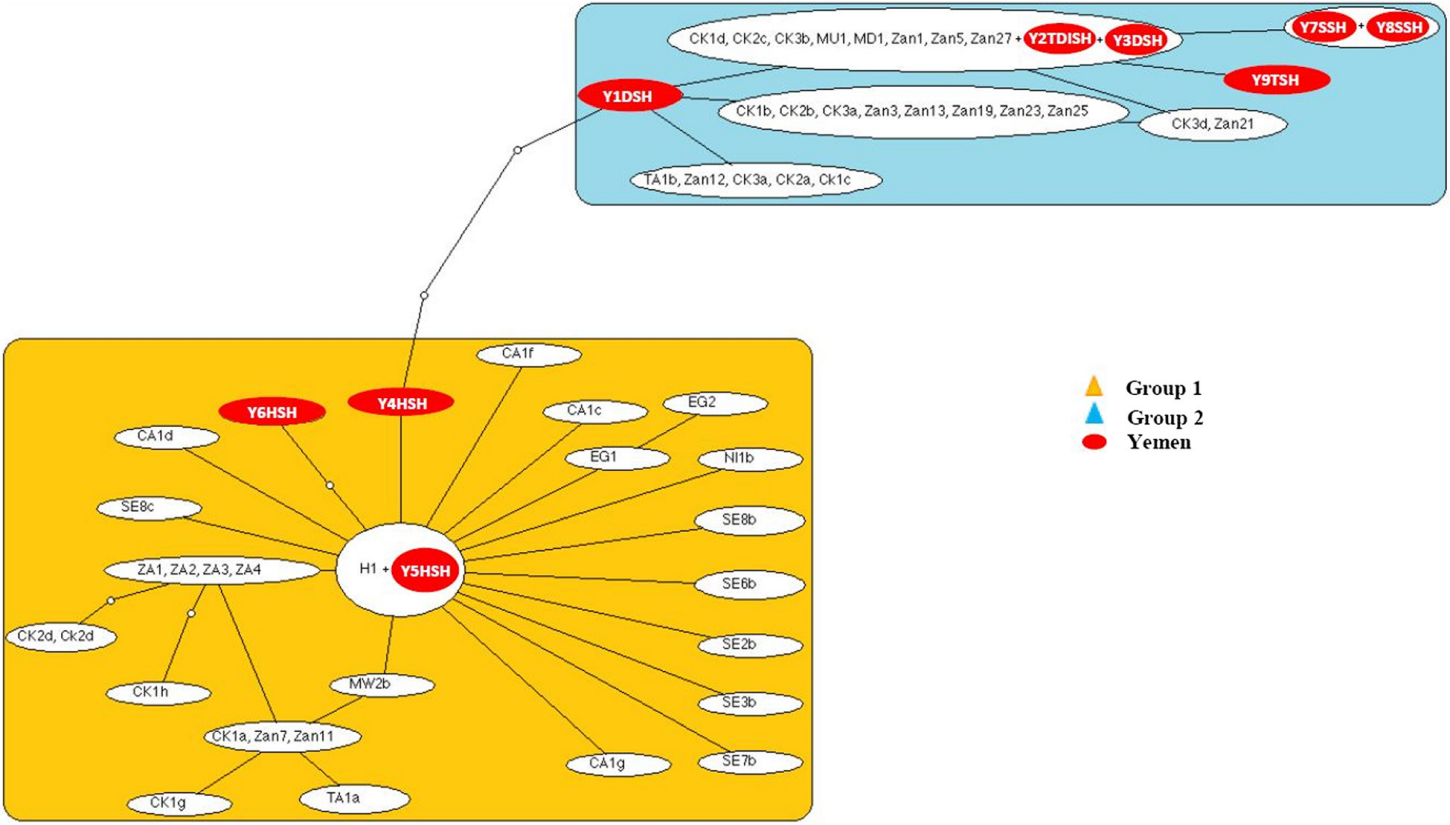
Minimum spanning TCS networks joining the 9 *S. haematobium* cox1 Yemeni haplotypes by this study and 18 countries across Africa and the Indian Ocean Islands from by Webster et al. [15, 22]. Each line between haplotypes represents a single bp change and small circles between lines represent unsampled or extinct haplotypes. H1 involved haplotypes from SE1, SE2a, SE3a, SE4, SE5, SE6a, SE7a, SE8a, SE9, MA2, NI1a, NI2, LB1, GB1, NG1, CA1, CA1a, CA2, CA3, CA4, CA5, SU1, KE2, TA1a, MW1, MW2a, MW3 & Zan4. Hodiedah haplotypes were exclusively linked with group 1 and Y5HSH was found similar to H1. The rest of Yemeni haplotypes were grouped with coastal Kenya, Zanzibar, Tanzania, Mauritius & Madagascar. Y2TDISH include Y2DSH, Y2TSH & Y2ISH haplotypes (group 2). Yemeni haplotypes linked the two groups of African haplotypes with small branches

Figure 5 shows the minimum spanning TCS network representing putative genealogy of the haplotypes at a locality level. The haplotypes divided into two groups. The first group (Group 1) was made up of 3 haplotypes all from Hodiedah while the second group (Group 2) was made up of 6 haplotypes from Taiz, Sana’a, Dhamar and Ibb. Both groups were linked via a long branch with several steps connecting the 2 haplotype groups. When the Yemen haplotypes were analysed together with the *S. haematobium cox*1 haplotype data from Webster et al. [22] the haplotypes were integrated into the 2 groups (Fig. 6). The most common haplotype found in Yemen grouped with the haplotypes found in Madagascar, Mauritius, Zanzibar and Coastal Kenya whilst 1 haplotype from Hodiedah matched the dominant mainland African haplotype H1. Five of the haplotypes from Yemen were also novel haplotypes which were not reported previously, 2 of which (Y1DSH and Y4HSH) actually formed a connection between the 2 groups.

### *Schistosoma haematobium* phylogenetic structuring

**Fig. 7:**
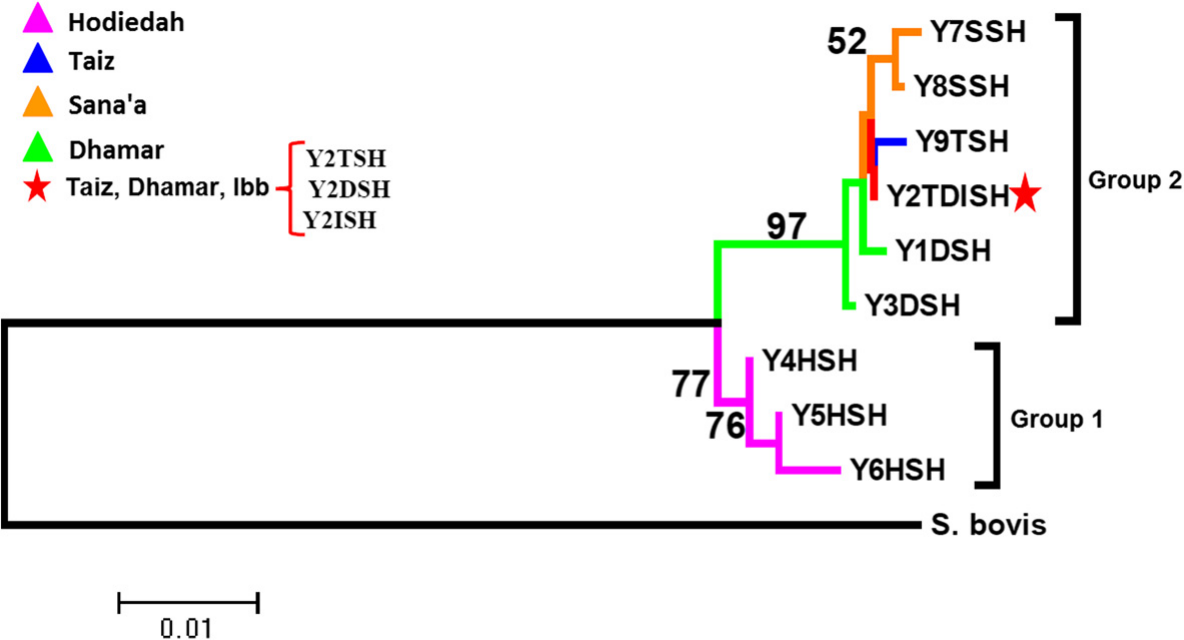
Neighbor-joining phylogenetic tree for *S. haematobium* with 1000 bootstrap values. Y2TDISH was the dominant haplotype detected in three provinces, Taiz (Y2TSH), Dhamar (Y2DSH), and Ibb (Y2ISH)

The tree topology supported the clustering of the 9 Yemen haplotypes into the two *S. haematobium* groups with the predominant haplotype (Y2TDISH) clustering within Group 2 together with haplotypes from Taiz, Ibb, Dhamar and Sana’a provinces, whereas the haplotypes from Hodiedah clustered with the Group 1 (Fig. 7). The net nucleotide divergence 0.01621 ± 0.00500 between the two *S. haematobium* groups was low compared to that between *S. haematobium* and is sister taxa *S. bovis*. (Group 1: 0.13252 ± 0.04940; Group 2: 0.12605 ± 0.05946).

### Neutrality and selection test

In this study a test for selection reinforced neutrality within the *cox1* mitochondrial data. Tajima’s D test and McDonald-Kreitman test results showed that strong selection was not occurring in either *S. mansoni* (Tajima’s D = 0.702; *P* > 0.10; Fisher’s exact test *P* = 0. 978) or *S. haematobium* (Tajima’s D = 0.747; *P* > 0.10; Fisher’s exact test *P* = 0.490).

## Discussion

Here we present the first genetic data on human *Schistosoma* species in Yemen. Schistosomiasis is still highly prevalent among children in rural Yemen [28] and 31.8 % of our cohort were infected with *Schistosoma* species. Out of these infected children, 122 (96.1 %) were found to be infected by either *S. mansoni* or *S. haematobium* while 5 (3.9 %) children were co-infected with both species. The overall prevalence was 23.8 % for *S. haematobium* and 9.3 % for *S. mansoni*. The highest overall prevalence of schistosomiasis was reported in Hodiedah province, followed by Taiz, while Dhamar had the lowest prevalence. Hodiedah had the highest prevalence of *S. haematobium*, while Ibb had the highest prevalence of *S. mansoni*.

We used conventional PCR to amplify the partial mt *cox*1 DNA region of *S. haematobium* and *S. mansoni* DNA extracted from stool and urine samples. The success rate using the PCR method to amplify *Schistosoma* DNA was 82.6 %, with some false negative reactions being attributed to errors in removing inhibitors from the samples. Moreover, additional processing required for the stool kit may also contribute to the low sensitivity of PCR when compared to microscopy. In the case of *S. haematobium*, this success rate was significantly associated with the number of eggs in the samples while no such association was found with *S. mansoni*.

From 31 *S. mansoni* PCR positive samples from 5 provinces, we obtained 19 unique and diverse haplotypes that divided into 4 lineages. These haplotypes give a good representation of *S. mansoni* diversity in Yemen however due to them being obtained from pooled samples there is more diversity yet to characterise. These 19 haplotypes integrated into groups 1, 2, 4 and 5 based on studies by Webster et al. [15] and Morgan et al. [21]. Of note, phylogenetic support was lower in some groups (1 and 4) when compared with previous reports. This may be due to the high number of haplotypes reported in this present study, and because of the use of a smaller *cox*1 DNA region (375 bp) being analysed in this study which decreases the number of parsimony informative positions. The larger the mt region used, the more haplotypes would be detected, but the geographic topologies of the data would remain the same [15]. Previous *cox*1 analyses of *S. mansoni* samples from across Africa have detected a high degree of genetic diversity within and between hosts and localities. Morgan et al. [21] discovered 85 unique haplotypes split into five lineages within 53 geographically widespread localities and Webster et al. [15] discovered 120 unique haplotypes split into five distinct lineages from 54 countries across South America, Africa and the Arabian Peninsula. Although lower numbers of *S. mansoni* samples were analysed from Yemen, the genetic diversity among *S. mansoni* haplotypes remained high strongly supporting the finding by Webster et al. [15] and Morgan et al. [21].

The TCS network and phylogenetic analysis show the high diversity of haplotypes that divided into 4 groups / lineages. The long connections between the main group (Group 1) and other groups were separated by nodes in the TCS network and were not represented by a detected genotype. This suggests that there are still more un-sampled haplotypes within and between the provinces. TCS analysis showed that central nodes were connected with other haplotypes, creating star like assemblages to form ancestral haplotypes, which were extensively abundant and widely distributed as suggested by Webster et al. [15]. In the current study, the ancestral haplotypes were found in Taiz and Ibb provinces in which, perhaps, the parasites spread to other provinces. Moreover, the highest genetic diversity was found among Ibb haplotypes, which were mostly distributed in Group 4, though Y6ISM was present in Group 1.

On the other hand, the genetic diversity of *S. mansoni* was found to be very low in Sana’a, Dhamar and Hodiedah probably attributed to the low prevalence of the *S. mansoni* in these provinces. The only haplotype of Hodiedah, found within Group 5, was linked with Dhamar haplotypes. Likewise, Ibb haplotypes linked the main ancestral haplotypes from Taiz within Group 1 with haplotypes of other provinces that showed lower genetic diversity. This is probably due to Ibb province being geographically connected with Taiz, Hodiedah and Dhamar provinces. It is important to mention that among the Yemeni population, Taiz and Ibb populations have the highest migration rate of people moving either to other Yemen provinces or other countries. These would suggest that Taiz and Ibb provinces are likely to be the origin of *S. mansoni* in Yemen due to the high genetic diversity found within those areas.

The net divergence between the lineages revealed a relatively short time span between the genetic separation of the Taiz, Ibb and Hodiedah (1, 4 and 5) lineages when compared to the larger divergence of lineage 2 consisting of Sana’a and Dhamar isolates. Moreover, the phylogenetic analysis showed a strong bootstrap for lineage 4, which involved Sana’a and Dhamar haplotypes, via a long branch from lineage 1. That said, there has been a long time between the separation of the haplotypes of Sana’a and Dhamar from the rest of the *S. mansoni* population, with successive splitting of populations within Ibb haplotypes in lineage 5. This may be attributed to population, movement, which carried those haplotypes between provinces, stating from Taiz to Ibb, then to Dhamar and ending with Sana’a province.

The results of the present study were incorporated with the results of the previous large-scale studies conducted on isolates from across Africa and also from the Arabian Peninsula and the Neotropic ecozone [15, 19]. Interestingly, for *S. mansoni*, our findings show that Yemen has a higher genetic diversity than Tanzania, Zambia and Coastal Kenya, which suggests that *S. mansoni* was first introduced in East Africa before spreading to Central and West Africa with subsequent splitting of populations. This is in accordance with a previous postulation that African *S. mansoni* evolutionary origin was in East Africa [21]. However, these speculations need further investigation as historical human migrations between Africa and Arabian Peninsula may have occurred continuously and reciprocally.

The regional TCS network for *S. mansoni* (Fig. 3) shows the isolates from Yemen may well bridge the gap between the African lineages. On the basis of this TCS network, one may speculate that Ibb haplotypes were probably introduced to Zambia and coastal Kenya (Group 4). On the other hand, other Ibb haplotypes (Group 1) were most probably moved to either Nigeria through a long branch to Central West Africa (Group 2), or Niger by a link to far West Africa (Group 1). In addition, Taiz haplotypes with a high genetic diversity were most properly moved to Saudi Arabia, which then links to both Brazil and Egypt.

With regard to *S. haematobium*, the present study shows that the genetic diversity of *S. haematobium* was low across Yemen, supporting the findings by Webster et al. [19] who revealed low levels of genetic diversity among 61 unique haplotypes from across Africa. In the 78 positive urine samples, we found only 9 unique haplotypes, which were divided into two groups. Group 2 involved 4 provinces, namely Sana’a, Taiz, Ibb and Dhamar, with Y2TDISH being the predominant haplotype. Whilst Group 1 exclusively involved haplotypes from Hodiedah. The net divergence between the two groups was similar to that previously reported in Webster et al. [19].

The TCS network shows that the predominant haplotype (Y2TDISH) was linked by a single bp change with other haplotypes from Taiz, Sana’a and two haplotypes from Dhamar, which connected Group 1 with Group 2. The majority of those haplotypes branched off from Y2TDISH by single mutations, although their clear links with other haplotypes suggest that they persist within the populations and disseminate from one area to another due to population movement. The network discovered around the predominant haplotype reflected the geographical links between the 4 provinces in Group 2, as well as the extensive movement of populations between those provinces. This TCS network suggests that Dhamar may be the origin of *S. haematobium* in Yemen, as this had the highest genetic diversity of all the provinces studied. However, this may need further investigation using more isolates including other provinces which were not included in the current study.

The divergence of the *S. haematobium* populations between the 2 groups might be affected by the compatibility with their intermediate snail hosts (*Bulinus spp.*), which are specific and varied according to geographical location [42, 43]. However, there have been no studies on the intermediate host of *S. haematobium* in Yemen, necessitating future research to elucidate the role of *Bulinus* species in the transmission of *S. haematobium* in Yemen.

The genetic diversity of *S. haematobium* in Yemen was considered high when compared with the low diversity across Africa, but not higher than the Indian Islands, and coastal Kenya regions [19]. The low genetic diversity reported across Africa was possibly attributed to a re-invasion by a small population of *S. haematobium* that originated as part of a larger population group from Asia across the Arabian Peninsula, with fast distribution and growth from East to West through Africa [19, 44]. Due to parasitic inbreeding, the worms are incompatible with the new genetic flow across Africa. While the TCS network created by Webster et al. [19] formed two distinct groups of *S. haematobium* haplotypes that cannot be linked, the TCS network generated by the present study shows Yemen haplotypes bridging the gap and connecting both groups, namely Group 1 (mainland Africa with few haplotypes from Zanzibar) and Group 2 (the Indian Ocean islands and the neighbouring African coastal regions of coastal Kenya and Tanzania) (Fig. 6). However, this will be a direct affect of the smaller DNA region used in the analysis which reduces the polymorphisms between the groups bringing them genetically closer.

This highly associated haplotype network between Yemen and Africa may be explained by the numerous commercial Yemeni traders trips that took place when sailors were using the monsoon winds to sail across the Indian Ocean, at which time they landed at the sheltered harbour located on the site of present-day Zanzibar Town in Tanzania. Although the islands had few resources of interest to the traders, they offered a good location from which to make contact and trade with towns of the East African coast. Nowadays, population movement between Yemen and East Africa including Kenya, Tanzania and Ethiopia is still very active. Another factor could be the annual Islamic pilgrimage that involved the travelling of people from all over the world to the Arabian Peninsula, specifically to Mecca. Although Mecca has an arid climate, which does not favour the transmission of schistosomiasis, the pilgrims would often cross through Yemen and other northern parts of the peninsula where schistosomiaisis was endemic.

This conjecture is supported by the discovery of the oldest ever urogenital *Schistosoma* egg in 6200-year-old human skeletal remains at a prehistoric town (Tell Zeidan) by the Euphrates river valley in northern Syria [45]. Moreover, modern genetic analysis suggests that the genus *Schistosoma* originally evolved in Asia and then spread to Africa [44]. There are a few theories on the origin of *Schistosoma*, with primary arguments being for both an African and Asian origin [46–48]. Davis [46] proposed that the genus *Schistosoma* arose before the separation of the super continent Gondwanaland more than 150 million years ago, with the earliest known archaeological examples coming from ancient Egyptian mummies and Syrian graves dated 5200 and 6500 years old respectively [45, 49].

In 2008, Yemen launched its first campaign to eliminate schistosomiasis as a national public health problem, with the aim of controlling schistosomiasis nationwide and eliminating its morbidity. This campaign was waged through repeated periodic (often yearly) distribution of PZQ to targeted population in schools and communities, together with the dissemination of health education messages on schistosomiasis [25]. Although PZQ has been used effectively for about three decades, the reliance of schistosomiasis control programmes on PZQ only makes the control of this disease highly vulnerable to the emergence and spread of drug resistant strains [9]. This might be anticipated due to drug pressure leading to intensive and prolonged new selection pressures on the parasites, which may in turn affect the genotypic and phenotypic structure of a parasite population in a controlled setting resulting in a decline in diversity over time to a few persistent genotypes [50, 51]. Non-susceptible survival genotypes with reduction of diversity were reported in a laboratory population [51, 52] and also among travellers in Egypt, Senegal and Mali [8, 53–55]. Furthermore, previous experience with oxamaniquine, which was in wide use prior to the development and use of PZQ, revealed the ability of schistosome parasites to develop drug resistance under field conditions [56, 57]. Hence, evolutionary theory must play a role both in the monitoring, evaluation, and importantly in predicting the sustained impact of these control programmes.

## Conclusion

The findings of the present study have brought new insight into the population genetics of human *Schistosoma* species across Yemen. In particular, the genetic diversity of *S. mansoni* was found to be high while *S. haematobium* showed low diversity. This data was obtained from pooled schistosome DNA from whole urine and stool samples. Therefore, by adapting the methods used by Webster et al. [19] which described obtaining the haplotype data from individual miracidia, the true extent of the diversity within and between *Schistosoma* populations can be fully assessed in future studies. Data compiled in order to identify genetic diversity as reported by this study, and in comparison with previous studies, may have a value in monitoring changes in schistosome populations over time, in response to control pressure and due to environmental changes or migration of hosts. The genetic analysis of more individual schistosome larval stages, together with investigations into intermediate host snails from different endemic areas in Yemen and other countries in the Arabian Peninsula, using other mitochondrial genes and microsatellite markers is still required in order to interpret the true genetic diversity, population movement and dynamics of transmission for *S. mansoni* and *S. haematobium* populations on a large scale.

## References

[1] Bruun B, Aagaard-Hansen J (2008). The social context of schistosomiasis and its control.

[2] Gryseels B (2012). Schistosomiasis. Infect Dis Clin North Am.

[3] World Health Organization (2013). Schistosomiasis: number of people treated in 2011. Wkly Epidemiol Rec.

[4] World Health Organization (1993). The control of schistosomiasis: second report of the WHO expert committee.

[5] Sesay S, Paye J, Bah MS, McCarthy FM, Conteh A, Sonnie M (2014). *Schistosoma mansoni* infection after three years of mass drug administration in Sierra Leone. Parasite Vector.

[6] Doenhoff MJ, Cioli D, Utzinger J (2008). Praziquantel: mechanisms of action, resistance and new derivatives for schistosomiasis. Curr Opin Infect Dis.

[7] Fallon PG, Doenhoff MJ (1994). Drug-resistant schistosomiasis: resistance to praziquantel and oxamniquine induced in *Schistosoma mansoni* in mice is drug specific. Am J Trop Med Hyg.

[8] Ismail M, Botros S, Metwally A, William S, Farghally A, Tao LF (1999). Resistance to praziquantel: direct evidence from *Schistosoma mansoni* isolated from Egyptian villagers. Am J Trop Med Hyg.

[9] Fenwick A, Webster JP (2006). Schistosomiasis: challenges for control, treatment and drug resistance. Curr Opin Infect Dis.

[10] Fenwick A, Webster JP, Bosque-Oliva E, Blair L, Fleming FM, Zhang Y (2009). The Schistosomiasis Control Initiative (SCI): rationale, development and implementation from 2002–2008. Parasitology.

[11] Stothard JR, French MD, Khamis IS, Basanez MG, Rollinson D (2009). The epidemiology and control of urinary schistosomiasis and soil-transmitted helminthiasis in schoolchildren on Unguja Island, Zanzibar. Trans R Soc Trop Med Hyg.

[12] Lamberton PHL, Hogan SC, Katbatereine NB, Fenwick A, Webster JP (2010). *In vitro* praziquantel test capable of detecting reduced *in vivo* efficacy in *Schistosoma mansoni* human infections. Am J Trop Med Hyg.

[13] Norton AJ, Gower CM, Lamberton PH, Webster BL, Lwambo NJ, Blair L (2010). Genetic consequences of mass human chemotherapy for *Schistosoma mansoni*: population structure pre- and post-praziquantel treatment in Tanzania. Am J Trop Med Hyg.

[14] Gower CM, Gabrielli AF, Sacko M, Dembele R, Golan R, Emery AM (2011). Population genetics of *Schistosoma haematobium*: development of novel microsatellite markers and their application to schistosomiasis control in Mali. Parasitology.

[15] Webster BL, Webster JP, Gouvras AN, Garba A, Lamine MS, Diaw OT (2013). DNA ‘barcoding’ of *Schistosoma mansoni* across sub-Saharan Africa supports substantial within locality diversity and geographical separation of genotypes. Acta Trop.

[16] Rollinson D, Webster JP, Webster B, Nyakaana S, Jørgensen A, Stothard JR (2009). Genetic diversity of schistosomes and snails: implications for control. Parasitology.

[17] Standley CJ, Kabatereine NB, Lange CN, Lwambo NJS, Stothard JR (2010). Molecular epidemiology and phylogeography of *Schistosoma mansoni* around Lake Victoria. Parasitology.

[18] Stothard JR, Webster BL, Weber T, Nyakaana S, Webster JP, Kazibwe F (2009). Molecular epidemiology of *Schistosoma mansoni* in Uganda: DNA barcoding reveals substantial genetic diversity within Lake Albert and Lake Victoria populations. Parasitology.

[19] Webster BL, Emery AM, Webster JP, Gouvras A, Garba A, Diaw O (2012). Genetic diversity within *Schistosoma haematobium*: DNA barcoding reveals two distinct groups. PLoS Negl Trop Dis.

[20] Betson M, Sousa-Figueiredo JC, Kabatereine NB, Stothard JR (2013). New insights into the molecular epidemiology and population genetics of *Schistosoma mansoni* in Ugandan pre-school children and mothers. PLoS Negl Trop Dis.

[21] Morgan JA, Dejong RJ, Adeoye GO, Ansa ED, Barbosa CS, Bremond P (2005). Origin and diversification of the human parasite *Schistosoma mansoni*. Mol Ecol.

[22] Webster BL, Culverwell CL, Khamis IS, Mohammed KA, Rollinson D, Stothard JR (2013). DNA barcoding of *Schistosoma haematobium* on Zanzibar reveals substantial genetic diversity and two major phylogenetic groups. Acta Trop.

[23] World Bank (2010). World Development Indicators 2010.

[24] Sady H, Al-Mekhlafi HM, Atroosh WM, Al-Delaimy AK, Nasr NA, Dawaki S (2015). Knowledge, attitude, and practices towards schistosomiasis among rural population in Yemen. Parasite Vector.

[25] Oshish A, AlKohlani A, Hamed A, Kamel N, AlSoofi A, Farouk H (2011). Towards nationwide control of schistosomiasis in Yemen: a pilot project to expand treatment to the whole community. Trans R Soc Trop Med Hyg.

[26] Abdulrab A, Salem A, Algobati F, Saleh S, Shibani K, Albuthigi R (2013). Effect of school based treatment on the prevalence of schistosomiasis in endemic area in Yemen. Iran J Parasitol.

[27] Al-Waleedi AA, El-Nimr NA, Hasab AA, Bassiouny HK, Al-Shibani LA (2013). Urinary schistosomiasis among schoolchildren in Yemen: prevalence, risk factors, and the effect of a chemotherapeutic intervention. J Egypt Public Health Assoc.

[28] Sady H, Al-Mekhlafi HM, Mahdy MA, Lim YA, Mahmud R, Surin J (2013). Prevalence and associated factors of Schistosomiasis among children in Yemen: implications for an effective control programme. PLoS Negl Trop Dis.

[29] Gray DJ, Ross AG, Li YS, McManus DP (2011). Diagnosis and management of schistosomiasis. BMJ.

[30] World Health Organization (2002). Prevention and control of schistosomiasis and soil-transmitted helminthiasis: report of a WHO expert committee. WHO Tech Rep Ser.

[31] Cheesbrough M (2009). District laboratory practice in Tropical Countries, part 1.

[32] Kosinski KC, Bosompem KM, Stadecker MJ, Wagner AD, Plummer J, Durant JL (2011). Diagnostic accuracy of urine filtration and dipstick tests for *Schistosoma haematobium* infection in a lightly infected population of Ghanaian schoolchildren. Acta Trop.

[33] Verweij JJ, Brienen EA, Ziem J, Yelifari L, Polderman AM, Van Lieshout L (2007). Simultaneous detection and quantification of *Ancylostoma duodenale*, *Necator americanus*, and *Oesophagostomum bifurcum* in fecal samples using multiplex real-time PCR. Am J Trop Med Hyg.

[34] Obeng BB, Aryeetey YA, de Dood CJ, Amoah AS, Larbi IA, Deelder AM (2008). Application of a Circulating-Cathodic-Antigen (CCA) strip test and real-time PCR, in comparison with microscopy, for the detection of *Schistosoma haematobium* in urine samples from Ghana. Ann Trop Med Parasitol.

[35] Webster BL, Rollinson D, Stothard JR, Huyse T (2010). Rapid diagnostic multiplex PCR (RD-PCR) to discriminate *Schistosoma haematobium* and *S. bovis*. J Helminthol.

[36] Van den Broeck F, Geldof S, Polman K, Volckaert FAM, Huyse T (2011). Optimal sample storage and extraction procotols for reliable multilocus genotyping of the human parasite *Schistosoma mansoni*. Infect Genet Evol.

[37] Thompson JD, Higgins DG, Gibson TJ (1994). CLUSTAL W: improving the sensitivity of progressive multiple sequence alignment through sequence weighting, position-specific gap penalties and weight matrix choice. Nucleic Acids Res.

[38] Kimura M (1980). A simple method for estimating evolutionary rates of base substitutions through comparative studies of nucleotide sequences. J Mol Evol.

[39] Tamura K, Peterson D, Peterson N, Stecher G, Nei M, Kumar S (2011). MEGA5: molecular evolutionary genetics analysis using maximum likelihood, evolutionary distance, and maximum parsimony methods. Mol Biol Evol.

[40] Posada D, Crandall KA (2001). Selecting the best-fit model of nucleotide substitution. Syst Biol.

[41] Librado P, Rozas J (2009). DnaSP v5: a software for comprehensive analysis of DNA polymorphism data. Bioinformatics.

[42] Standley CJ, Goodacre SL, Wade CM, Stothard JR (2014). The population genetic structure of *Biomphalaria choanomphala* in Lake Victoria, East Africa: implications for schistosomiasis transmission. Parasite Vector.

[43] Rollinson D, Stothard JR, Southgate VR (2001). Interactions between intermediate snail hosts of the genus Bulinus and schistosomes of the *Schistosoma haematobium* group. Parasitology.

[44] Lawton SP, Hirai H, Ironside JE, Johnston DA, Rollinson D (2011). Genomes and geography: genomic insights into the evolution and phylogeography of the genus *Schistosoma*. Parasite Vector.

[45] Anastasiou E, Lorentz KO, Stein GJ, Mitchell PD (2014). Prehistoric schistosomiasis parasite found in the Middle East. Lancet Infect Dis.

[46] Davis GM (1992). Evolution of prosobranch snails transmitting Asian *Schistosoma*; coevolution with *Schistosoma*: a review. Prog Clin Parasitol.

[47] Morgan JA, Dejong RJ, Snyder SD, Mkoji GM, Loker ES (2001). *Schistosoma mansoni* and Biomphalaria: past history and future trends. Parasitology.

[48] Webster BL, Southgate VR, Littlewood DTJ (2006). A revision of the interrelationships of *Schistosoma* including the recently described *Schistosoma guineensis*. Int J Parasitol.

[49] Kloos H, David R (2002). The paleoepidemiology of schistosomiasis in ancient Egypt. Hum Ecol Rev.

[50] Feng Z, Curtis J, Minchella DJ (2001). The influence of drug treatment on the maintenance of schistosome genetic diversity. J Math Biol.

[51] Coeli R, Baba EH, Araujo N, Coelho PMZ, Oliveira G (2013). Praziquantel treatment decreases *Schistosoma mansoni* genetic diversity in experimental infections. PLoS Negl Trop Dis.

[52] Rogers SH, Bueding E (1971). Hycanthone resistance: development in *Schistosoma mansoni*. Science.

[53] Doenhoff MJ, Hagan P, Cioli D, Southgate V, Pica-Mattoccia L, Botros S (2009). Praziquantel: its use in control of schistosomiasis in sub Saharan Africa and current research needs. Parasitology.

[54] Fallon PG, Sturrock RF, Niang AC, Doenhoff MJ (1995). Short report: diminished susceptibility to praziquantel in a Senegal isolate of *Schistosoma mansoni*. Am J Trop Med Hyg.

[55] Grandière-Pérez L, Ansart S, Paris L, Faussart A, Jaureguiberry S, Grivois JP (2006). Efficacy of praziquantel during the incubation and invasive phase of *Schistosoma haematobium* schistosomiasis in 18 travelers. Am J Trop Med Hyg.

[56] Bonesso-Sabadini PI, de Souza Dias LC (2002). Altered response of strain of Schistosoma mansoni to oxamniquine and praziquantel. Mem Inst Oswaldo Cruz.

[57] Valentim CL, Cioli D, Chevalier FD, Cao X, Taylor AB, Holloway SP (2013). Genetic and molecular basis of drug resistance and species-specific drug action in schistosome parasites. Science.

